# Therapeutics and Materia Medica

**Published:** 1874-12

**Authors:** 


*Therapeutics and Materia Medica. A systematic Treatise on the action and uses of Medicinal Agents, including their Description and History.* By Alfred Stillé, M. D., Prof. etc. Philadelphia: Henry C. Lea, 1874. Buffalo: T. Butler & Son.

Stillé's Therapeutics and Materia Medica has been so long and favorable known by the profession, that it seems unnecessary for us to make an extended notice of its contents. Since the publication of the last edition the work has been thoroughly revised, and in a large measure rewritten. The additions to the work have increased its size by about two hundred and fifty pages, and comprise a consideration of several new articles of Materia Medica besides additions to those comprised in the last edition. The classification employed in the first edition is still used. Medicinal agents are divided into twelve great classes, comprising Lenitives, Astringents, Irritants, Tonics, General Stimulants, Cerebro-Spinal Stimulants, Spinants, General Sedatives, Arterial Sedatives, Nervous Sedatives, Evacuants and Alteratives.

The first volume embraces the introduction, which is a general consideration of medicines, their action and mode of administration, and the first six classes of remedial agents. Volume two completes the consideration of the remaining six classes, and also contains a copious index both to the Materia Medica and to Therapeutics.

As this work has heretofore been the standard American treaties on Materia Medica, so it will continue to be regarded. Prof. Stillê has disappointed many who looked for the appearance of his work long ago, but they can easily overlook their disappointment when they consider how well he has used the time during which they were compelled to wait.

